# China launched a pilot project to improve its rare disease healthcare levels

**DOI:** 10.1186/1750-1172-9-14

**Published:** 2014-01-27

**Authors:** Yazhou Cui, Xiaoyan Zhou, Jinxiang Han

**Affiliations:** 1 Shandong Academy of Medical Science, Shandong Medical Biotechnological Center, Key Laboratory for Biotech Drugs of the Ministry of Health, Jinan 250062, China

## Abstract

China is facing the great challenge of serving the world’s largest rare disease population. It is necessary to develop a specific medical plan to increase the levels of optimal prevention, diagnosis and treatment of rare diseases under the existing clinical service structures in China. In 2013, China launched its first pilot project focused on 20 representative rare diseases. A national network including approximately 100 provincial or municipal medical centers has been established to enable collaboration on rare diseases across China. The main objectives for this project are to develop and apply medical guidelines and clinical pathways for rare diseases, to establish a rare disease patient registry and data repository system, and to promote molecular testing for rare genetic disorders. This project also emphasizes building close links among the collaborative network, clinicians on the frontlines in basic medical services institutions and rare disease patient organizations. Primarily, this project expects to develop an actionable medical services plan to increase the delivery of quality healthcare for individuals and families living with rare diseases in China within five years.

## Background

China’s is facing the great challenge of servicing the world’s largest rare disease population. Although several medical strategies critical to discovery and treatment of rare diseases have been implemented in China, such as newborn screening [1], and medical expenses reimbursement for children with congenital heart disease and leukemia [2], the Chinese public health insurance system does not cover the specific medical requirement of most rare diseases patients nor internationally recognized orphan drugs due to a lack of legislation.

For the most part, Western countries, which have orphan drug or rare disease legislation with mandates for research and services, also have some plan of action and/or management of rare diseases. While this does not lead to uniform healthcare services throughout these countries, there are not the great disparities in terms of geographic regions and hospital levels, as there is in China [3]. This makes it difficult for China to closely model many aspects of the rare disease healthcare systems implemented in Western countries. Therefore, exploration of the status quo, potential implementation of known models, and the creation of new models, is urgently needed to increase optimal prevention, timely diagnosis, and quality treatment of rare diseases in China in the context of the current healthcare system and beyond.

There are three great challenges in improving healthcare for rare diseases in China. First, a general lack of information about these conditions, and rare diseases in general, often leads to delayed diagnosis and misdiagnosis or inappropriate treatments for a significant proportion of patients with rare disease. Medical guidelines and clinical pathways are valuable for patients with rare diseases in optimizing clinical outcomes and maximizing clinical efficiency. The Ministry of Health (MOH) of China began to launch the pilot implementation of clinical pathways from December 2009, and nearly half of China’s public-funded hospitals have implemented the clinical pathways. Up to February 2013, the MOH have released 331 clinical pathways for diseases under 24 disciplines (http://www.ch-cp.org.cn/?action-zhengcejiedu). However, most of these guidelines are designed for common and high incidence diseases; only 46 clinical pathways (13.9%) were developed for rare diseases. Furthermore, these clinical pathways still have problems in recording, editing, and statistics [4]. Secondly, a general lack of patient registry and data repository systems limits the initiation of epidemiologic studies and multi-center clinical trials on rare diseases. And the lack of important epidemiological data and registered clinical cases has severely hampered the introduction of national laws dealing with rare diseases [5]. Third, the causative gene has been discovered for approximately 80% of rare diseases. Currently, genetic tests are usually unavailable for most of the patients with rare genetic diseases and their doctors because of the scarcity of molecular diagnostic resources and their associated high costs in China. In a Chinese literature review on genetic skeletal diseases, genetic mutation testing was performed in only a small portion of patients (187 of 16,099 cases, 1.16%) by several university hospitals, and then only for research objectives [6].

To address the rare disease care challenges delineated above, in 2013, China launched its first pilot national-level project to promote the advancement of rare disease healthcare levels. To conduct this project, a national collaborate network involving more than 100 provincial and municipal medical centers was established by China Rare Diseases Prevention and Treatment Alliance. This network covers 13 provinces, which have a population of 0.7 billion.

### The frames of the project

This project selected 20 representative rare diseases as pilot diseases including arrhythmogenic right ventricular cardiomyopathy, congenital myotonia, congenital pyriform sinus fistula, Duchenne and Becker muscular dystrophy, epidermolysis bullosa, Fahr syndrome, familial aortic aneurysm, hereditary spastic paraplegia, left ventricular noncompaction, Marfan syndrome, myotonic dystrophy, neurofibromatosis, osteogenesis imperfecta, primary tethered spinal cord syndrome, primary cardiac sarcomas, pseudoxanthoma elasticum, Sturge-Weber syndrome, thoracic aortic aneurysm and dissection, tuberous sclerosis complex, and Wilson Disease. The selection was based on the following characteristics: 1) typical rare diseases with low prevalence but not extremely rare; 2) life-threatening or chronically debilitating but not classified as inadequately treated diseases; and 3) scarcity of experienced physicians specialized in the treatment of these rare diseases except in very few medical centers across the country, and no corresponding medical guideline and clinical pathway has been proposed.

The first aim of this project is to develop and pilot-test medical guidelines and clinical pathways for 20 example rare diseases. In particular, the project will focus on enforcing polycentric pilot-tests for medical guidelines and clinical pathways of rare diseases, developing standard models and templates to improve associated or emerging checklists, and prioritizing items for further evaluation. The project is first committed to organizing experienced medical centers specializing in the abovementioned 20 rare diseases and to establishing the corresponding medical guidelines and clinical pathways. In the second stage, these rare diseases medical guidelines and clinical pathways will be pilot-tested in approximately 100 provincial or municipal medical centers within the national collaborative network. During the pilot testing, the criteria will be modified to maximize the agreement and adoption between hospitals. Then, the revised medical guidelines and clinical pathways will be submitted to committees of experts from the MOH and Chinese Medical Association for review to be further applied in hospitals nationwide.

The second objective of this project is to establish a patient registry and data repository for 20 example rare diseases through the national rare diseases network, including more than 100 provincial and municipal medical centers. The inpatient medical records of these medical centers from 2003 to 2012 will be first retrospectively reviewed to identify cases of the 20 example rare diseases from the past 10 years. Then, newly diagnosed example rare diseases cases will be prospectively registered from 2013–2016. A data repository of de-identified patient data will be aggregated in a standardized manner, using Common Data Elements (CDEs) and standardized terminology. In addition, we have developed a web-based open-source patient registry system that will be released to the public, as a service to the patient organizations and others, to allow and encourage them to establish additional rare disease patient registries.

Third, this project aims to establish a supporting molecular genetic testing center for rare diseases. Next-generation sequencing (NGS) technologies could offer fast, comprehensive and cost-efficient genetic screening for genetic diseases and have been successfully adopted in the genetic diagnostics of some rare diseases with genetic heterogeneity, such as cardiomyopathies [7,8]. In an attempt to increase the utility of gene tests in the diagnosis and treatment of Chinese rare genetic disorders, in this project, the pilot supporting molecular genetic testing center is critical component in advancing better care for individuals and families. Initially, nine single gene and seven NGS-based panel analyses covering 15 example rare diseases will be developed to support molecular genetic diagnostic services.

### Challenges in implementing the project

Because of their scarcity, it is often difficult for frontline doctors to identify individual rare diseases conditions in China, especially the doctors in primary hospitals (< 100 bed) and secondary hospitals (>100 but <500 bed), which are mainly responsible for treating rural residents and account for more than 70% of the medical resources in China. This is not unlike a challenge in the United States where more than 86% of patients are seen in community centers and not in specialty centers. In Europe, there is a delay in diagnosis for 50% of patients, even though centers of excellence exist for the diseases surveyed [9]. Therefore, bridging the gap between frontline clinicians and our collaborated network represents a large challenge in implementing this project. To improve this situation, the project will launch a national continuing education campaign to increase knowledge and awareness of rare diseases for frontline doctor in low-level medical resources. The campaign will primarily focus on the abovementioned 20 example rare diseases.

Patient organizations and their members play an active and instrumental role in improving the healthcare levels of rare diseases. Recently, several patient organizations have been established, but they are still limited. The project is also committed to exploring strategies to support more patient organizations in China through our national collaborative rare diseases network, with a particular focus on example rare diseases.

### Expected outcomes and future prospects

Within the frame of the project, a coordinated and structural network for rare diseases at the country level could be established. Improvements in example rare disease healthcare levels will be assessed. The strengthening of genetic testing services together with the establishment of medical guidelines and clinical pathways should enable the diagnosis of the pilot diseases of interest and appropriate treatment within a reasonable period of time. Analysis of data from the rare disease patient registry and repository system would be helpful for evaluating the prevalence, regional distribution and expense of rare diseases, which will provide an important reference for health authorities when drafting medical policies on rare diseases and orphan drugs. This project is also committed to increasing the recognition of rare diseases in the public and doctors at low-level hospitals. We hope a prevention and treatment pattern suitable for China can be developed by this explorative project. In the long term, we will look for methods to scale up and apply the medical system to 200 to 300 rare diseases over the next five years. Chinese experience and strategies will also provide references to other countries when they confront their own rare disease healthcare challenges.

## Competing interests

The authors declare that they have no competing interests.

## Authors’ contributions

JH and YC conceived and designed the study. YC was responsible for the manuscript writing. XZ contributed to the design of the study. All authors read and approved the final manuscript.
